# Investigating the Therapeutic Effects of Naringenin and Oleuropein on Prostate Cancer Cell Mat-LyLu via miR-155-5p: A Bioinformatics and Molecular Docking Analysis of *KRAS* and *CDK2* Networks

**DOI:** 10.3390/genes17010079

**Published:** 2026-01-09

**Authors:** Cigdem Gungormez

**Affiliations:** Department of Medical Biology, Faculty of Medicine, Siirt University, 56100 Siirt, Turkey; gungormezcigdem@gmail.com

**Keywords:** prostate cancer cells, miR-155, oleuropein, naringenin, molecular docking, network pharmacology

## Abstract

**Background:** This study systematically investigates the therapeutic effects of naringenin (NAR) and oleuropein (OLE) on prostate cancer through miR-155-5p regulation. **Methods:** Experimental studies conducted on MAT-LyLu prostate cancer cell lines revealed that the application of NAR (50 μM) and OLE (75 μM) significantly increased miR-155-5p expression by 2.89-fold and 1.74-fold, respectively (*p* < 0.05). Bioinformatics analyses have indicated that miR-155-5p interacts with critical oncogenic pathways such as *KRAS*, *CDK2*, NF-κB, and TGF-β/Smad2. Computational analyses have revealed that miR-155-5p interacts with 16 critical oncogenic targets, including *KRAS* and *CDK2.* Molecular docking studies showed that NAR binds to the Switch I/II region of *KRAS* with a binding energy of −8.2 kcal/mol, while OLE binds to the ATP-binding pocket of *CDK2* with an affinity of −9.1 kcal/mol. Pharmacokinetic evaluations revealed that NAR indicated high oral bioavailability (93.763% HIA) and full compliance with Lipinski’s rules, while OLE required advanced formulation strategies due to its high polarity. Network pharmacology analyses have shown that NAR affects lysosomal functions and enzyme regulation, while OLE affects G protein-coupled receptors and oxidoreductase activity. **Results**: Results indicate that NAR and OLE exhibit antitumor effects through multiple mechanisms by increasing miR-155-5p expression and inhibiting critical oncogenic targets in prostate cancer. **Conclusions:** Findings suggest that the dietary intake of these natural compounds (citrus and olive products) should be considered in prostate cancer prevention strategies, shedding light on the epigenetic mechanisms of polyphenols in cancer treatment and contributing to the development of new therapeutic strategies.

## 1. Introduction

Prostate cancer (PCa) is the second most commonly diagnosed malignancy worldwide after lung cancer, posing a significant global health issue [1,2]. Although significant progress has been made in understanding PCa pathogenesis, genetic predisposition, ethnic differences, geographical differences and environmental factors contribute to the development of the disease, its full etiology remains unclear [3,4]. However, potential treatments for the cancer are still being investigated.

Despite the existence of accumulated clinical strategies for the management of PCa, there is a lack of prognostic/sensitive biomarkers that can monitor the onset and progression of the disease. Additional markers are therefore needed [5,6]. MicroRNAs (miRNAs) are endogenous, non-coding RNA molecules 18–24 nucleotides in length that play a critical role in the post-transcriptional regulation of gene expression [7]. These molecules interact with mRNA with low specificity, regulating gene expression through mRNA degradation and translational inhibition mechanisms [8,9]. In particular, in prostate cancer (PCa), abnormal miRNA expression profiles have been shown to directly contribute to the carcinogenesis process [10]. Due to their pleiotropic effects, miRNAs hold potential for use as biomarkers in elucidating the molecular mechanisms of PCa, as well as offering promise in the development of early diagnosis, prognostic assessment, and treatment strategies.

miR-155-5p is a miRNA that is actively post-transcriptionally regulated in many types of cancer. Its overexpression in prostate cancer, in particular, has been shown in various studies to modulate transcriptional connections with many oncogenic and tumor suppressor genes [11,12]. Cai et al. demonstrated that miRNA-155 supports the proliferation of prostate cancer cells by regulating ANX7 expression levels [13].

Advances in molecular science have shed light on the structural basis of the anti-cancer effects of plants [14]. Polyphenols are phytochemicals that have attracted particular interest due to their aromatic structures and antioxidant, anti-inflammatory, and anti-tumor properties [15,16]. These compounds have emerged as promising anticancer agents for prostate cancer chemoprevention [17,18,19]. The genomic and epigenomic modulation capabilities of polyphenols enhance their potential to prevent carcinogenesis [20]. Chemoprevention strategies are attracting research interest for their complementary role to traditional treatments.

Naringenin (citrus) and oleuropein (olive) are powerful bioactive polyphenols. With their antioxidant, anti-inflammatory, and anticarcinogenic properties, they exhibit therapeutic potential in both nutraceuticals and pharmaceutical applications [21,22]. These compounds demonstrate antitumor effects through multiple mechanisms, including tumor invasion inhibition, apoptotic pathway activation, and regulation of oncogenic miRNA networks [23,24]. However, the exact mechanisms of naringenin (NAR) and oleuropein (OLE) in prostate cancer, particularly through miRNA modulation, have not been fully elucidated. Current bioinformatics approaches (miRNA target analysis, network pharmacology, and molecular docking simulations) enable systematic investigation of these mechanisms [25,26,27].

miR-155-5p is a pleiotropic and context-dependent microRNA whose biological function varies according to cancer type, disease stage, and cellular microenvironment. Although miR-155 is frequently described as an oncogenic microRNA in prostate cancer, increasing evidence indicates that miR-155-5p may also exert tumor-suppressive functions under specific conditions, particularly through the negative regulation of metastatic and inflammatory signaling pathways such as TGF-β/SMAD2, NF-κB, and SPOCK1. Importantly, compound- or stress-induced modulation of miR-155-5p may activate compensatory negative feedback mechanisms that attenuate oncogenic signaling rather than reflect its basal oncogenic overexpression. Therefore, the functional role of miR-155-5p in prostate cancer should be interpreted in a context-dependent manner rather than as uniformly oncogenic or tumor suppressive.

In this study, an integrated approach combining experimental validation with computational modeling is used to elucidate the molecular interactions between NAR, OLE, and miR-155-5p in prostate cancer cells, identify potential therapeutic targets, and characterize binding interactions. The goal is to provide critical information for developing new chemopreventive strategies.

## 2. Materials and Methods

### 2.1. Data Collection and miRNA Specification

miRNA expression profiles associated with prostate cancer were obtained from the NCBI Gene Expression Omnibus (GEO) database (http://www.ncbi.nlm.nih.gov/geo, accessed on 30 July 2024). This study utilized the GSE189208 (4 samples), GSE86917 (8 samples), and GSE109356 (12 samples) datasets, with all data generated using the Affymetrix Human Genome U133-Plus-2.0 platform (Applied Biosystems, Thermo Fisher Scientific, Waltham, MA, USA) and miRNA expression analyses were performed using the Agilent Human 0.6K miRNA Microarray (G4471A) platform (Agilent, Santa Clara, CA, USA). Differential expression analyses were performed using GEO2R, comparing normal samples with prostate cancer samples of different Gleason scores. Significantly differentially expressed genes (adj. *p* < 0.05, FDR-corrected, and |logFC| > 1 criteria) were identified, and miR-155-5p consistently emerged as the most significantly deregulated miRNA across all array results, leading to further studies focused on this miRNA.

### 2.2. Cell Culture and Compound Application Protocol

In previous studies, Aktas and Akgün (2018) investigated the concentration-dependent effects of naringenin on Mat-LyLu cells in their study of the Nav1.7 voltage-gated sodium channel and found that 75 μM inhibited proliferation, while 5–10 μM reduced migration [28]. Similarly, Aktas and Ayan (2020) reported the time- and dose-dependent anti-proliferative effects of oleuropein (25–50 μM) [29]. Based on the current data, 50 μM naringenin and 75 μM oleuropein concentrations, which do not exhibit cytotoxic effects, were selected to investigate the potential regulatory effects of these compounds on the miRNA-155-5p expression profile. The MAT-LyLu cell line was provided by the Ion Channels and Cancer Research Laboratory of the Biology Department of Harran University. The highly metastatic Dunning model rat prostate cancer cell line MAT-LyLu was cultured in RPMI-1640 complete medium (1% FBS, 2 mM L-glutamine, 250 nM dexamethasone) under standard culture conditions (5% CO_2_, 37 °C, 95% humidity) and passaged to propagate. The compound naringenin, a compound with a molecular weight of 272.25 g/mol, was dissolved in dimethyl sulfoxide (DMSO) to prepare a 10 mM stock solution, which was stored at +4 °C. Oleuropein, a compound with a molecular weight of 540.51 g/mol, was filtered through a 0.2 μm sterile filter and dissolved in RPMI-1640 to prepare a 10 mM stock solution. Media containing 50 μM naringenin and 75 μM oleuropein were applied to cells in the log phase. After controlled incubation (5% CO_2_, 37 °C, 48 h), the cells were washed three times with PBS. The pellets were resuspended in Qiazol and stored at −80 °C until isolation. The experiments were performed in triplicate.

### 2.3. Evaluation of the Effect of OL and NAR on miRNA Expression Levels

Total RNA isolation from MAT-LyLu cells removed with Qiazol was performed using the miRNeasy Mini Kit (Qiagen, Hilden, Germany) in accordance with the manufacturer’s protocol. The purity and concentration of the extracted RNA were checked by evaluating the 260/280 and 260/230 absorption ratios in spectrophotometric measurements and by visualizing it with an electrophoresis gel. For quantitative analysis, 100 ng of total RNA was reverse transcribed using the Qiagen miRCURY LNA RT Kit. miR-155-5p (MIMAT0030409) expression levels were determined using the miRCURY LNA SYBR Green PCR Kit on the Bio-Rad CFX96 qPCR system and normalized using the 2^−ΔΔCt^ method by comparing with the reference gene U6 small nuclear RNA. All experiments were performed in *n* = 5 independent biological replicates, and statistical analyses were performed using the Student’s *t*-test in GraphPad Prism 10.5.0 (GraphPad Software, San Diego, CA, USA) software, with *p* < 0.05 considered statistically significant. Data normality was assessed prior to Student’s *t*-test.

### 2.4. DIANA Target Gene Identification

The DIANA Tools miRPath v.3 (http://diana.imis.athena-innovation.gr/DianaTools/index.php, accessed on 30 July 2024) integrated database was used for the systematic analysis of miR-155-5p’s molecular targets and related biological pathways. Expression changes in Mat-LyLu cells treated with naringenin and oleuropein were subjected to pathway enrichment analysis using the Kyoto Encyclopedia of Genes and Genomes (KEGG) (https://www.genome.jp/kegg/pathway.html, accessed on 30 July 2024) prostate cancer pathway (hsa05215) as a reference. These analyses were designed to reveal the potential effects of these compounds on cancer-related signaling cascades through the regulation of target genes by miR-155-5p.

### 2.5. Gene Association Network Analysis

To systematically evaluate the functional role of miR-155-5p in prostate cancer, target gene annotation protein interaction analysis was performed using the KEGG prostate cancer pathway map (hsa05215) with DIANA mirPath v.3 software. The disease-target pathway (D-P) network created as a result of the pathway analyses was integrated with compound-target (C-D) interaction data to form a comprehensive compound-disease-pathway (C-D-P) interaction network. Based on these analyses, protein–protein interaction (PPI) analyses were performed for 16 critical regulatory genes (*KRAS*, *CDK2*, *RELA*, *EGFR*, *E2F2/3*, *CCND1*, *CDKN1A*, *NFKB1*, *CTNNB1*, *PIK3R1*, *BCL2*, *GSK3B*, *FOXO1*, *AKT3*, and *NKX3-1*) were visualized using protein–protein interaction (PPI) analyses with the STRING database v11.5 (https://string-db.org/, accessed on 30 July 2024) (minimum interaction score > 0.7), and the functional connections of these genes in prostate cancer pathogenesis were revealed.

### 2.6. In Silico Analysis of Pharmacokinetic Properties and Drug Similarity

A comprehensive evaluation of the pharmacokinetic profiles and drug similarity of naringenin (PubChem CID: 439246) and oleuropein (PubChem CID: 5281544) was performed using multiple in silico tools. The SMILES formats of the compounds are [Naringenin: OC1=CC=C(C=C1)C(C2)OC3=CC(O)=CC(O)=C3C2=O; Oleuropein: COC(=O)C1=CO[C@@H](O[C@@H]2O[C@H](CO)[C@@H] (O)[C@H](O)[C@H]2O)\C(=C\C)[C@@H]1CC(=O)OCCc3ccc(O)c(O)c3] were uploaded to the SwissADME platform (https://www.swissadme.ch/, accessed on 10 August 2024) and analyzed using standard parameters. These analyses revealed comprehensive pharmacokinetic properties, including compliance with Lipinski’s rule, gastrointestinal absorption, blood–brain barrier permeability, and drug similarity scores. Additionally, the ADMETLAB2 platform (https://admetmesh.scbdd.com/, accessed on 10 August 2024) was used to systematically evaluate the ADMET properties, toxicological parameters, and therapeutic index values of both compounds.

### 2.7. Molecular Docking and Target Prediction Analyses

The molecular interactions between the central target proteins *KRAS* (PDB ID: 6GJ7) and *CDK2* (PDB ID: 1HCL) identified in the protein–protein interaction network analysis and Naringenin and Oleuropein were comprehensively analyzed using the SwissDock platform (http://www.swissdock.ch/, accessed on 20 August 2024). In this study, the 3D structures of the compounds obtained from the PubChem database (NAR: PubChem CID 439246, OLE: PubChem CID 5281544) were converted to PDB format by energy minimization using UCSF Chimera 1.15. Simultaneously, high-probability biological target prediction analyses were performed for NAR and OLE using SwissTargetPrediction (http://www.swisstargetprediction.ch/, accessed on 10 August 2024).

## 3. Results

### 3.1. Effect of Naringenin and Oleuropein on miR-155-5p Expression

**Figure 1:**
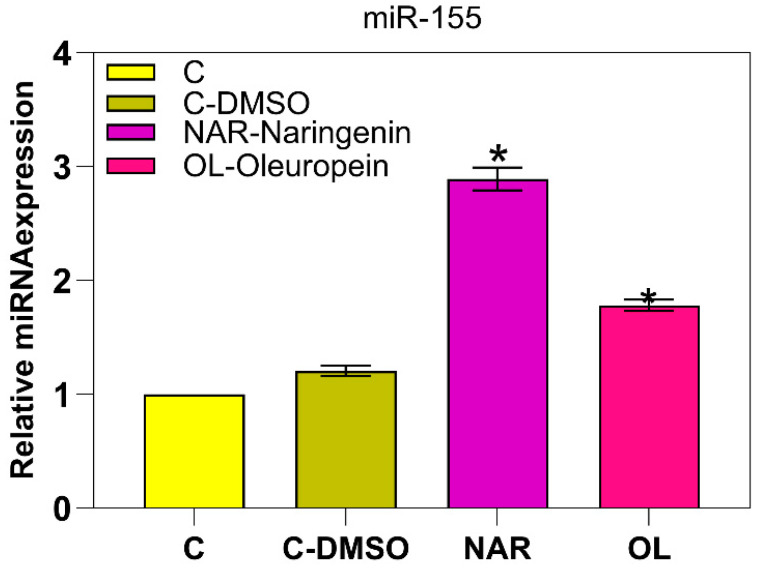
miR-155-5p expression fold change: Control; C-DMSO: Control-DMSO; NAR: Naringenin; OLE: Oleuropein (* *p* < 0.05).

To investigate the effect of naringenin (NAR) and oleuropein (OLE) on miR-155-5p expression, MAT-LyLu prostate cancer cells were treated with NAR (50 μM) and OLE (75 μM) for 24 h. Quantitative real-time PCR analysis showed that both compounds significantly increased miR-155-5p expression compared to untreated control cells (*p* < 0.05). Specifically, NAR treatment resulted in a 2.89-fold increase, whereas OLE treatment induced a 1.74-fold increase in miR-155-5p expression (Figure 1). These data indicate that NAR and OLE are capable of modulating miR-155-5p expression in MAT-LyLu cells. However, these results describe expression changes only and do not directly imply a tumor-suppressive or oncogenic function of miR-155-5ps.

### 3.2. miRNA Target Interactions

**Figure 2:**
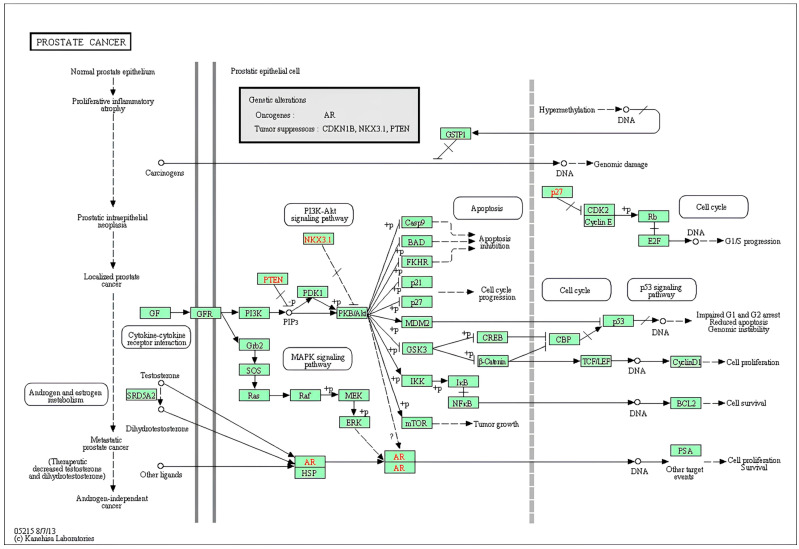
KEGG pathway prostate cancer (KEGG hsa05215).

Network pharmacology analysis was performed to identify putative molecular targets and pathways associated with NAR and OLE. The predicted targets were enriched in pathways related to enzyme regulation, signal transduction, and cell cycle–associated processes. To elucidate the functional effects of miR-155-5p upregulation, a comprehensive pathway analysis was performed using the computational framework of DIANA Tools. This integrated platform combines multiple validated algorithms (miRBase v22, TargetScan 7.2, and microT-CDS) and carefully selected databases to systematically analyze miRNA-mRNA interactions and disease associations. Through DIANA mirPath v.3, the predicted gene targets of miR-155-5p in the prostate cancer pathway (KEGG hsa05215) were specifically queried. Analysis parameters included target gene binding energy < −20 kcal/mol, seed region (2–8 nt) full complementarity, and pathway impact score > 0.5 criteria. Statistically significant results obtained using Fisher’s test of precision suggest that miR-155-5p binding sites are significantly enriched in various oncogenic signaling cascades involved in cell proliferation, evasion of apoptosis, and metastatic progression, particularly in the PI3K-AKT and MAPK signaling pathways (Figure 2). These analyses are predictive and exploratory in nature and aim to provide a framework for hypothesis generation rather than direct experimental validation.

### 3.3. Gene Association Network Analysis Results

**Figure 3:**
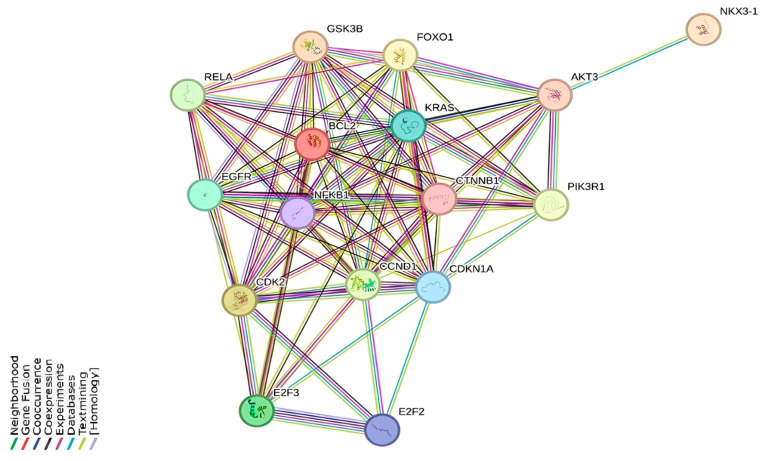
Showing the miRNA-associated protein–protein interaction (PPI) network and its possible main targets for Mat-LyLu cells treated with OLE and NAR.

To systematically investigate the functional relationships among miR-155-5p target genes, protein–protein interaction (PPI) network analysis was performed using the STRING database (version 11.5). This comprehensive platform created biologically meaningful interaction networks by combining multiple types of evidence, including gene co-expression patterns, genetic interactions, experimentally validated protein–protein interactions, and annotated pathway descriptions. Our analysis focused on 16 central genes (*KRAS*, *CDK2*, *RELA*, *EGFR*, *E2F3*, *E2F2*, *CCND1*, *CDKN1A*, *NFKB1*, *CTNNB1*, *PIK3R1*, *BCL2*, *GSK3B*, *FOXO1*, *AKT3*, *NKX3-1*). The resulting PPI network suggested extensive connections among these central genes and enrichment in key oncogenic pathways such as cell cycle regulation (*CDK2*, *CCND1*, *CDKN1A*), proliferative signaling (*KRAS*, *EGFR*, *AKT3*), and apoptosis modulation (*BCL2*, *FOXO1*) (Figure 3). The high level of network connectivity among these miR-155-5p targets suggests coordinated regulation of multiple oncogenic pathways and provides a mechanistic basis for the anti-proliferative effects observed following naringenin and oleuropein treatment.

### 3.4. Drug-Likeness Prediction, Pharmacokinetics and Toxicity of Oleuropein and Narıngenin

The pharmacokinetic properties of NAR and OLE were evaluated using the SwissADME platform http://www.swissadme.ch/, accessed on 10 December 2024) to provide supportive information regarding their physicochemical and absorption-related characteristics. Naringenin demonstrated favorable oral bioavailability and compliance with Lipinski’s rule of five, whereas oleuropein showed limitations associated with molecular size and polarity. These data are presented as complementary pharmacokinetic information and do not imply direct therapeutic efficacy. NAR demonstrated lower polarity and structural complexity with an sp^3^ hybrid carbon ratio (Fsp3) of 0.133 and a topological polar surface area (TPSA) of 86.990 Å^2^, while oleuropein exhibited significantly higher polarity (TPSA = 201.670 Å^2^) and structural complexity (Fsp3 = 0.520).

**Figure 4:**
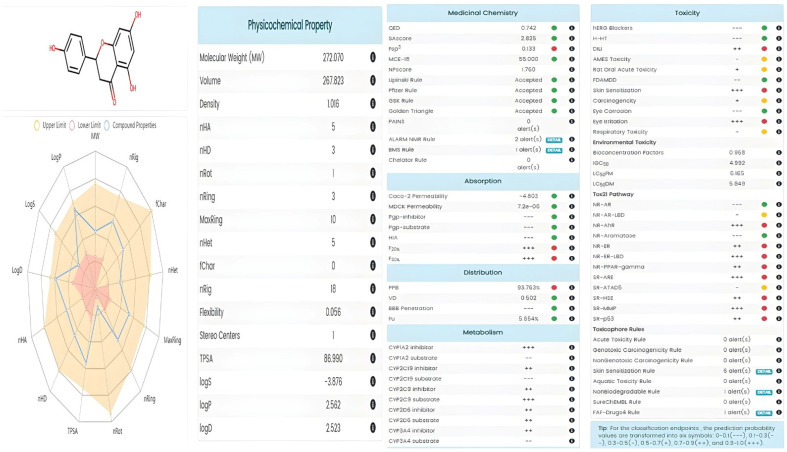
Naringenin pharmacokinetic properties.

**Figure 5:**
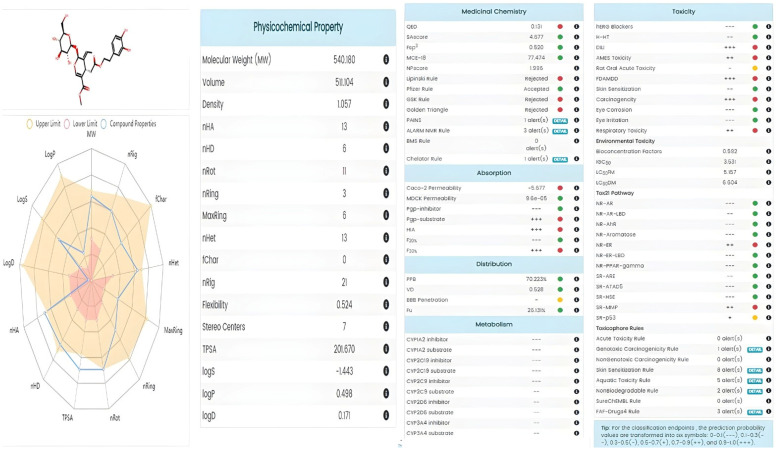
Oleuropein pharmacokinetic properties.

Drug similarity assessments revealed that NAR met all of Lipinski’s five rules, Pfizer’s three/five rules, and GSK’s 4/400 criteria, indicating the compound’s high oral bioavailability potential (Figure 4). In contrast, OLE only met Pfizer’s criteria, with limited compliance identified in other drug similarity guidelines due to its high molecular weight and polarity (Figure 5).

Toxicity profiling revealed that NAR was within the safe range for all toxicity parameters except for mild eye irritation, while oleuropein required additional toxicological evaluation. These findings indicate that NAR may be a more suitable candidate for pharmaceutical applications. The BOILED-Egg predictive model demonstrated favorable absorption characteristics for both compounds (Figure 4 and Figure 5).

In silico ADME analyses were comprehensively examined to evaluate the pharmaceutical potential of NAR and OLE in the BOILED Egg model. NAR stands out due to its high gastrointestinal absorption (93.763% HIA), blood–brain barrier permeability, and lack of P-gp substrate properties, making it a promising candidate for oral administration and central nervous system-targeted therapeutic strategies (Figure 4). Additionally, its full compliance with Lipinski’s and other drug similarity rules indicates high bioavailability potential. Oleuropein, on the other hand, exhibits a relatively limited absorption profile due to its higher polarity and molecular weight; however, it is believed that these limitations could be overcome through structural optimization or advanced delivery systems.

### 3.5. Swissdock Analysis Result Pharmacokinetic and Physicochemical Properties

**Figure 6:**
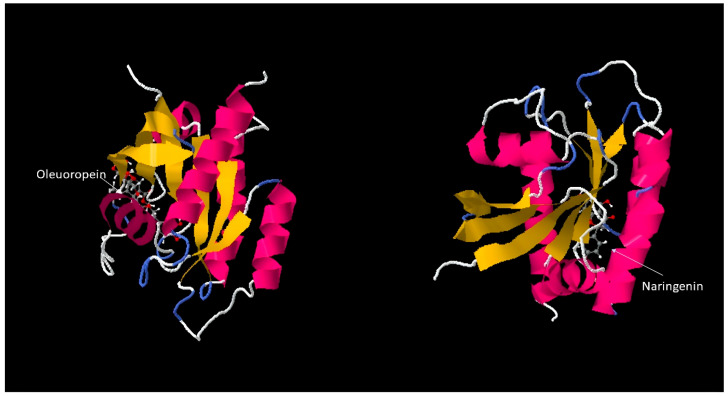
Docking of NAR and OLE for *KRAS*.

**Figure 7:**
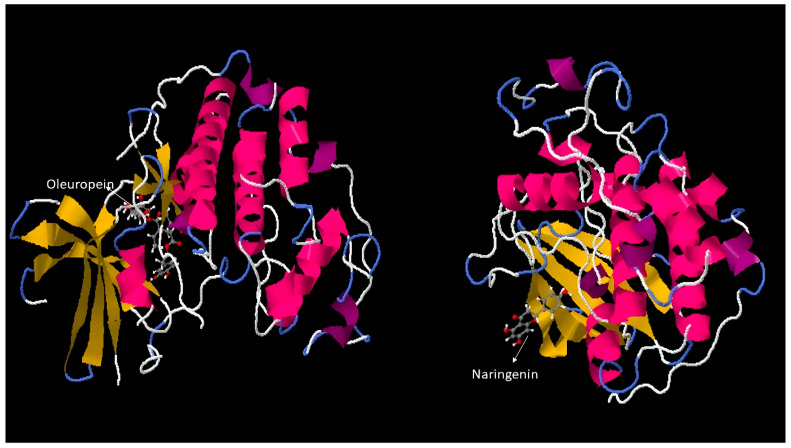
Docking of Oleuropein and Naringenin for *CDK2*.

Molecular docking simulations were conducted to predict potential binding interactions between the investigated compounds and selected target proteins. Following target identification using DIANA and STRING analyses, the binding conformations of NAR and OLE with two critical oncogenic targets, *KRAS* (Figure 6) and *CDK2* (Figure 7), were examined. Docking results showed that both compounds effectively bound to the active regions (shown in yellow) of these target proteins. Specifically, NAR formed stable interactions with key residues in the GTP-binding pocket of *KRAS*, while OLE exhibited strong binding affinity to the ATP-binding region of *CDK2*. The predicted binding energies and molecular orientations suggest that these compounds may act as competitive inhibitors and potentially interfere with the normal oncogenic activities of proteins. These computational findings provide structural insights into how NAR and OLE may exert their anticancer effects at the molecular level, particularly through direct modulation of cell cycle regulation (via *CDK2* inhibition) and proliferative signaling pathways (via *KRAS* interaction). These findings suggest possible compound–target interactions at the structural level; however, they represent computational predictions and require experimental validation.

**Figure 8:**
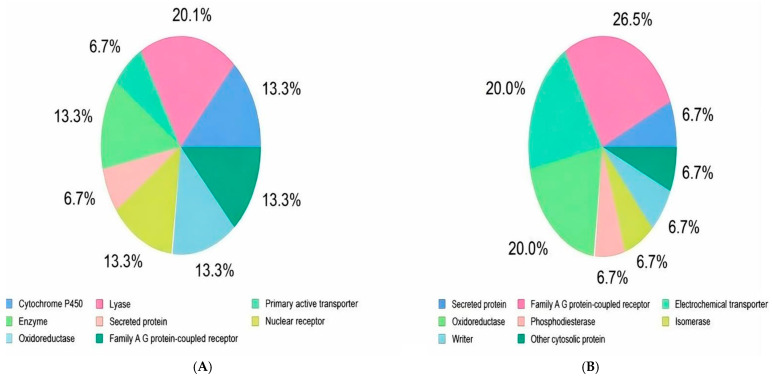
(**A**) Naringenin biological process effect (**B**) Oleuropein biological process effect.

Comprehensive network pharmacology analyses have indicated that NAR and OLE can exhibit different modulatory effects on multiple biological targets. NAR showed significant activity, particularly on lyase functions (20%) and enzyme regulation (13.3%), and exhibited meaningful interactions with G protein-coupled receptors (A Family) and primary active transporters. OLE, similarly multifaceted but with a distinct pharmacological profile, exhibited strong interactions with G protein-coupled receptors (26.7%), oxidoreductase activity (20%), and electrochemical transport systems (Figure 8).

These findings suggest that while both compounds possess pleiotropic effect mechanisms, they may exert their protective effects through partially distinct molecular pathways. In particular, the 20% activity rates in lyase and oxidoreductase systems indicate that these phytochemicals may play a critical role in the metabolic and epigenetic regulation of cancer cells. The high interaction rate of oleuropein with G protein-coupled receptors (26.7%) reveals its potential to modulate intracellular signaling pathways that are frequently disrupted in prostate cancer. The target profiles obtained are consistent with the characteristic multi-target interaction properties of polyphenol-derived compounds, reflecting their capacity to simultaneously modulate multiple critical nodes in oncogenic signaling networks. These network-based findings are consistent with our experimental data and molecular mapping results, supporting the potential use of these compounds as multi-targeted therapeutic agents in prostate cancer. In particular, the simultaneous inhibition of metabolic reprogramming and signaling pathways in cancer cells may offer significant strategic advantages in preventing treatment resistance.

## 4. Discussion

This study suggests that naringenin (NAR) and oleuropein (OLE) may exert antiproliferative and regulatory effects in prostate cancer cells, and that this may be regulated by modulating miR-155-5p-mediated *KRAS*/*CDK2* targets. Considering NAR’s high oral bioavailability and OLE’s synergistic effect, the intake of these compounds through diet (citrus fruits, olives, and olive oil) or standardized supplements may be recommended as a chemopreventive strategy. In particular, the Mediterranean diet’s rich content of these compounds may provide a significant advantage in protecting against prostate cancer. In pharmaceutical applications, while NAR has the potential for direct oral use, advanced formulation strategies such as nanoparticle carrier systems should be developed for OLE. Future preclinical and clinical studies are critical for elucidating the dose optimization, pharmacodynamic effects, and role of these compounds in combination therapies, as well as validating their therapeutic efficacy. Furthermore, although our docking studies provide valuable information about potential drug-target interactions, in vitro and in vivo validation is required to confirm these predictions. The bioavailability and pharmacokinetic properties of NAR and OLE also require further research to evaluate their clinical applicability. Among the limitations of our study are the need for in vivo validation to confirm the clinical translation potential of the in vitro model, the need to validate changes in the expression of miR-155-5p target genes using Western blot or ELISA, and the need for a more detailed investigation of the time-dependent pharmacodynamic effects of the compounds.

This study explores the regulatory role of miR-155-5p in prostate cancer cells using an integrated experimental and computational approach. Our findings suggest the potential of NAR and OLE as multi-targeted candidates for further investigation in prostate cancer. In this study, treatment with NAR (50 μM) and OLE (75 μM) resulted in a significant upregulation of miR-155-5p expression (2.89-fold and 1.74-fold, respectively). These results indicate that both compounds are capable of modulating miR-155-5p expression; however, the biological consequences of this modulation should be interpreted in a context-dependent manner.

The role of miR-155-5p in prostate cancer is complex and highly context-dependent. While miR-155 has frequently been described as an oncogenic microRNA and reported to be overexpressed in prostate cancer and other malignancies, increasing evidence demonstrates that miR-155-5p can also exert inhibitory effects on metastatic and inflammatory signaling pathways under specific cellular conditions [13,30,31,32]. Several studies have shown that miR-155-5p negatively regulates key components of the TGF-β/SMAD2, NF-κB, and SPOCK1 pathways, leading to the suppression of migration and invasion rather than enhanced proliferation [32,33,34,35]. In particular, Guolong et al. demonstrated that selenium nanoparticles inhibited prostate cancer metastasis through a miR-155-5p–mediated negative feedback mechanism involving NF-κB and SMAD2 signaling [32]. Similarly, Lin et al. reported that miR-155-5p reduced the metastatic potential of lung adenocarcinoma cells by targeting SMAD2, while Yao et al. identified SPOCK1 as a direct downstream target of miR-155-5p. These findings highlight the dual and context-dependent nature of miR-155-5p function in cancer biology.

In the present study, the observed increase in miR-155-5p expression following NAR and OLE treatment is interpreted as a compound-induced regulatory response rather than a reflection of basal oncogenic overexpression. Such induction may represent a compensatory negative feedback mechanism that modulates oncogenic signaling in highly metastatic prostate cancer cells [33]. Nevertheless, given the pleiotropic and context-dependent behavior of miR-155-5p, the functional implications of its upregulation require cautious interpretation and further experimental validation.

Flavonoids such as NAR and OLE are well recognized for their pleiotropic biological activities, including antioxidant, anti-inflammatory, and anticancer effects [36,37,38,39]. Previous studies have shown that oleuropein alters the expression of multiple microRNAs associated with apoptosis and immune regulation [40], while NAR has been reported to modulate miRNA expression linked to the PI3K–Akt signaling pathway [41]. These findings collectively support the concept that flavonoid-induced miRNA modulation may contribute to the regulation of cancer-related signaling networks. Accordingly, our results suggest that NAR and OLE may influence prostate cancer–related pathways through context-dependent modulation of miR-155-5p rather than through a single, linear mechanism.

Our molecular docking analyses further support this hypothesis by demonstrating that NAR is predicted to bind to the Switch I/II regions of *KRAS*, while oleuropein shows favorable binding affinity toward the ATP-binding pocket of *CDK2*. These interactions suggest a potential for indirect modulation of proliferative and cell cycle–related signaling pathways. From a network pharmacology perspective, the distinct target profiles of NAR (lyase functions and enzyme regulation) and OLE (G protein–coupled receptors and oxidoreductase activity) are consistent with the characteristic multi-target effects of polyphenolic compounds. Such pleiotropic interactions may provide a strategic advantage in overcoming treatment resistance through simultaneous modulation of multiple oncogenic nodes.

*CDK2* is a key regulator of cell cycle progression, and its dysregulation has been implicated in prostate cancer development and progression [42,43]. Previous studies have emphasized CDK2 as a potential therapeutic target, and our docking results suggest that NAR and OLE may interact with this kinase. Similarly, *KRAS*, a frequently altered oncogene in prostate adenocarcinoma, represents another potential regulatory node influenced by miRNA-mediated mechanisms [44,45]. Although our docking and network analyses support these interactions at a predictive level, experimental validation is required to confirm their functional relevance.

From a pharmaceutical perspective, SwissADME analyses indicated that NAR exhibits favorable oral bioavailability and drug-likeness properties, whereas oleuropein may require formulation optimization to overcome bioavailability limitations. Taken together, these findings suggest that NAR may be prioritized for oral chemopreventive strategies, while advanced delivery systems such as nanoparticle-based formulations could enhance the therapeutic potential of OLE.

Several limitations of this study should be acknowledged. The lack of functional validation experiments, including target gene expression analysis and gain- or loss-of-function studies for miR-155-5p, limits the mechanistic conclusions that can be drawn. Another limitation of this study is the use of a single rat-derived prostate cancer cell line, which warrants further validation in human prostate cancer models. Future studies should aim to address these limitations to better define the therapeutic relevance of miR-155-5p modulation by NAR and OLE.

## Data Availability

The original contributions presented in this study are included in this article. Further inquiries can be directed to the corresponding author.
